# Combined tubular adenocarcinoma, neuroendocrine carcinoma and adenocarcinoma with enteroblastic differentiation arising in Barrett esophagus

**DOI:** 10.1007/s12328-023-01791-0

**Published:** 2023-04-07

**Authors:** Kotaro Sugawara, Takashi Fukuda, Yutaka Kishimoto, Daiji Oka, Yoshiyuki Kawashima, Naoko Inoshita, Hiroaki Kanda

**Affiliations:** 1 Department of Gastroenterological Surgery, Saitama Cancer Center Hospital, Saitama, Japan; 2 Department of Pathology, Moriyama Memorial Hospital, Tokyo, Japan; 3 Department of Pathology, Saitama Cancer Center Hospital, 780 Komuro Inamachi, Kitaadachi-Gun, Saitama, 362-0806 Japan

**Keywords:** Barrett esophagus, Esophageal adenocarcinoma, Enteroblastic, Neuroendocrine carcinoma

## Abstract

Adenocarcinoma (AC) with neuroendocrine carcinoma (NEC) or enteroblastic (ENT) differentiation rarely develops in Barrett’s esophagus (BE). A 76-year-old man was diagnosed with Barrett’s AC (cT1bN0M0) and underwent thoracoscopic esophagectomy. A type 0-IIc + 0-Is lesion measuring 26 × 21 mm was macroscopically observed on a background of long segment BE (pT1bN0M0). The tumor comprised three different histological types of carcinoma (NEC, AC with ENT differentiation and moderately differentiated AC). NEC showed positivity for synaptophysin, chromogranin A and insulinoma-associated protein 1 with a Ki-67 index of 60.6%. ENT tumors were immunopositive for AFP and sal-like protein 4, and focally immunopositive for human chorionic gonadotrophin. The amounts of NEC, ENT and AC were 40%, 40% and 20%, respectively. p53 expression was positive throughout the tumor. Rb expression was negative at the NEC, but positive at the ENT and AC. CD4 and CD8 densities were lower in the NEC segment than in the AC and ENT segments, and PD-L1 expression was negative throughout the tumor. Early cancer arising in BE with a combination of tubular AC, ENT tumors and NEC is very rare. Our observations might contribute to understanding the carcinogenetic pathways and tumor microenvironment of NEC and ENT tumors.

**Supplementary Information:**

The online version contains supplementary material available at 10.1007/s12328-023-01791-0.

## Introduction

Barrett’s esophagus (BE), which is characterized by the replacement of normal esophageal squamous cell epithelium with columnar metaplasia, affects approximately 1% of the global population and increases the risk of developing esophageal carcinoma (EC) [1]. Most neoplasms arising in BE are tubular adenocarcinoma (AC) [2], while other tissue types, such as neuroendocrine carcinoma (NEC) or adenocarcinoma with enteroblastic (ENT) differentiation, are rare in BE [3–5]. These rare tumors reportedly exhibit more aggressive biology than AC [6, 7]. However, due to rarity, their histopathological features and immune profiles have yet to be fully investigated [8]. Herein, we document a very rare case with a tumor arising in BE, which was comprised of tubular AC, AC with ENT differentiation, and NEC.

## Case presentation

A 76-year-old man visited our facility for detailed examination of a flat protruding lesion in the esophagogastric junction that had been detected by esophagogastroduodenoscopy during a medical examination. The patient had a history of hypertension and early prostate cancer. He had long experienced symptoms associated with gastroesophageal reflux disease, and had taken proton-pump inhibitors. The blood examination results were normal except for chronic renal dysfunction (eGFR 42.4). Serum levels of several tumor markers were elevated; alpha-fetoprotein (AFP) (19.6 ng/ml, normal range < 10 ng/ml), carbohydrate antigen 19–9 (40 U/ml, normal range < 37 U/ml) and pro-gastrin-releasing peptide (103.6 pg/ml, normal range < 67 pg/ml). Other tumor markers, such as carcinoembryonic antigen (CEA), neuron-specific enolase and anti-p53-antibody, were within their normal ranges.

**Fig. 1:**
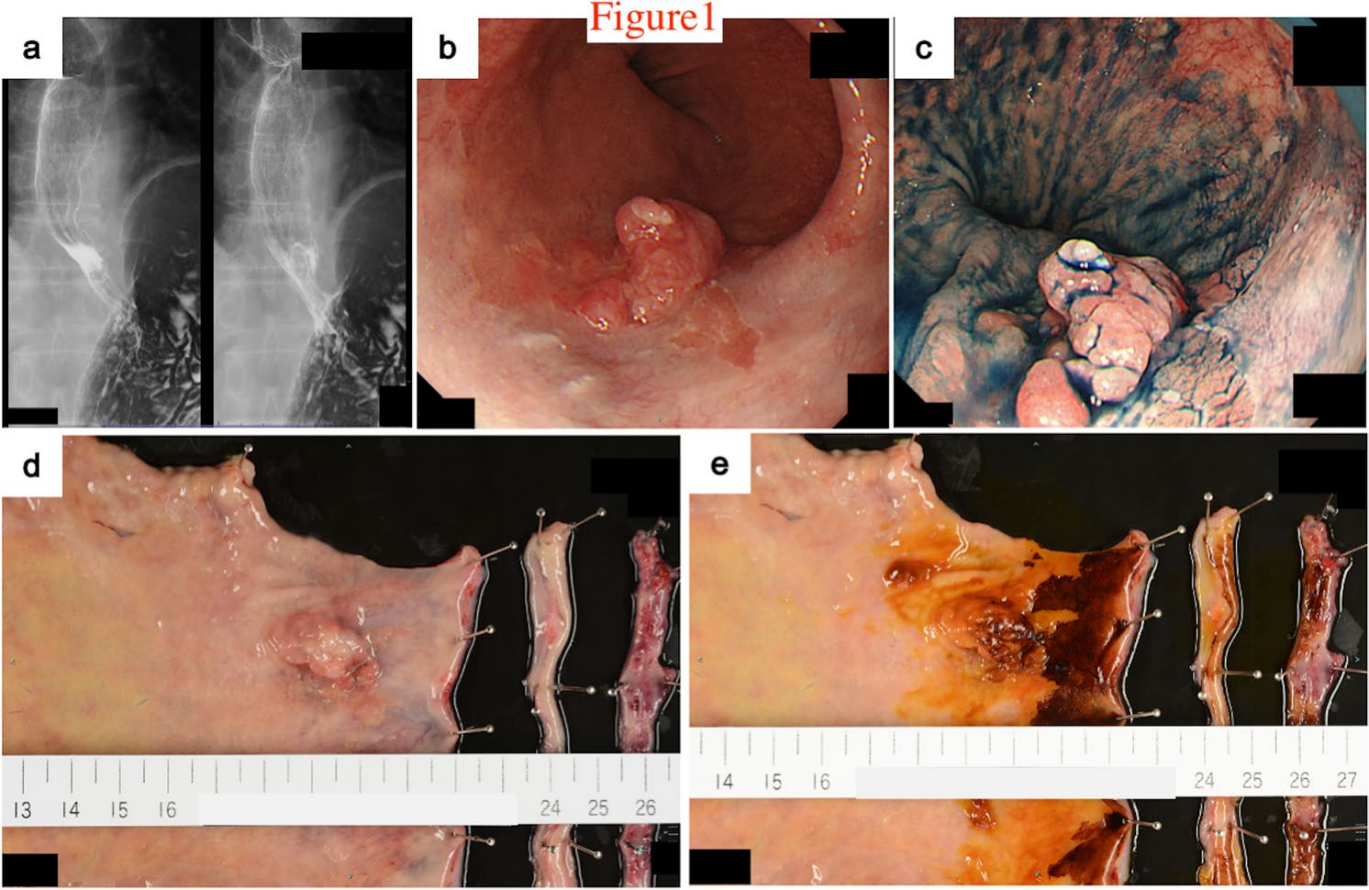
Esophagography and endoscopic findings before surgery, and in the resected specimen. **a** Esophagography findings. A type 0-IIc + 0-Is elevated lesion was found in the abdominal esophagus. **b**, **c** Upper gastrointestinal endoscopy detected a protruding 20 mm lesion (type 0-IIc + 0-Is) on the posterior wall of the esophagogastric junction. The lesion was on a background of long segment Barrett’s esophagus. **d** A type 0-IIc + 0-Is lesion measuring 26 × 21 mm was macroscopically observed at the squamous-columnar junction. **e** Lugol’s iodine staining

Esophagography revealed a type 0-IIc + 0-Is elevated lesion in the abdominal esophagus, with a size of 20 mm, that showed apparent invasion into the submucosa (Fig. 1a). Esophagogastroduodenoscopy confirmed a flat, erythematous, slightly protruding 20 mm lesion (type 0-IIc + 0-Is) on the posterior wall of the esophagogastric junction (Fig. 1b, c). The proximal end of the lesion did not reach the white normal squamous epithelium. The lesion was observed to be on a background of long segment BE, in which columnar-appearing mucosa was accompanied by palisade vessels. Pathologic evaluation of the biopsy specimen indicated portions of the tubular adenocarcinoma, which was covered with normal stratified squamous epithelium. Neither distant metastasis nor lymph node involvement was seen on thoracoabdominal contrast computed tomography. The patient was diagnosed with Barrett's adenocarcinoma, classified as clinical stage I (T1bN0M0) according to the 8th TNM classification [9].

The patient was treated by thoracoscopic middle to inferior mediastinal lymph node dissection, proximal gastrectomy, distal esophagectomy and reconstruction with jejunal interposition. He was discharged from the hospital 21 days after the surgery, without complications.

### Histopathological findings

In the resected specimen, a type 0-IIc + 0-Is lesion measuring 26 × 21 mm was macroscopically observed at the squamous-columnar junction (Fig. 1d). Staining with Lugol’s iodine revealed that part of the flat protruding lesion was covered by squamous epithelium (Fig. 1e). Histological examination of the resected specimens revealed some islands of squamous epithelium, esophageal glands beneath the columnar epithelium, and double-layered muscularis mucosae, suggesting the neoplastic lesion to be within BE. The background mucosa of BE mainly comprised of the cardiac-type mucosa, and intestinal-type mucosa was slightly recognized nearby the oral margin of the tumor [12].

**Fig. 2:**
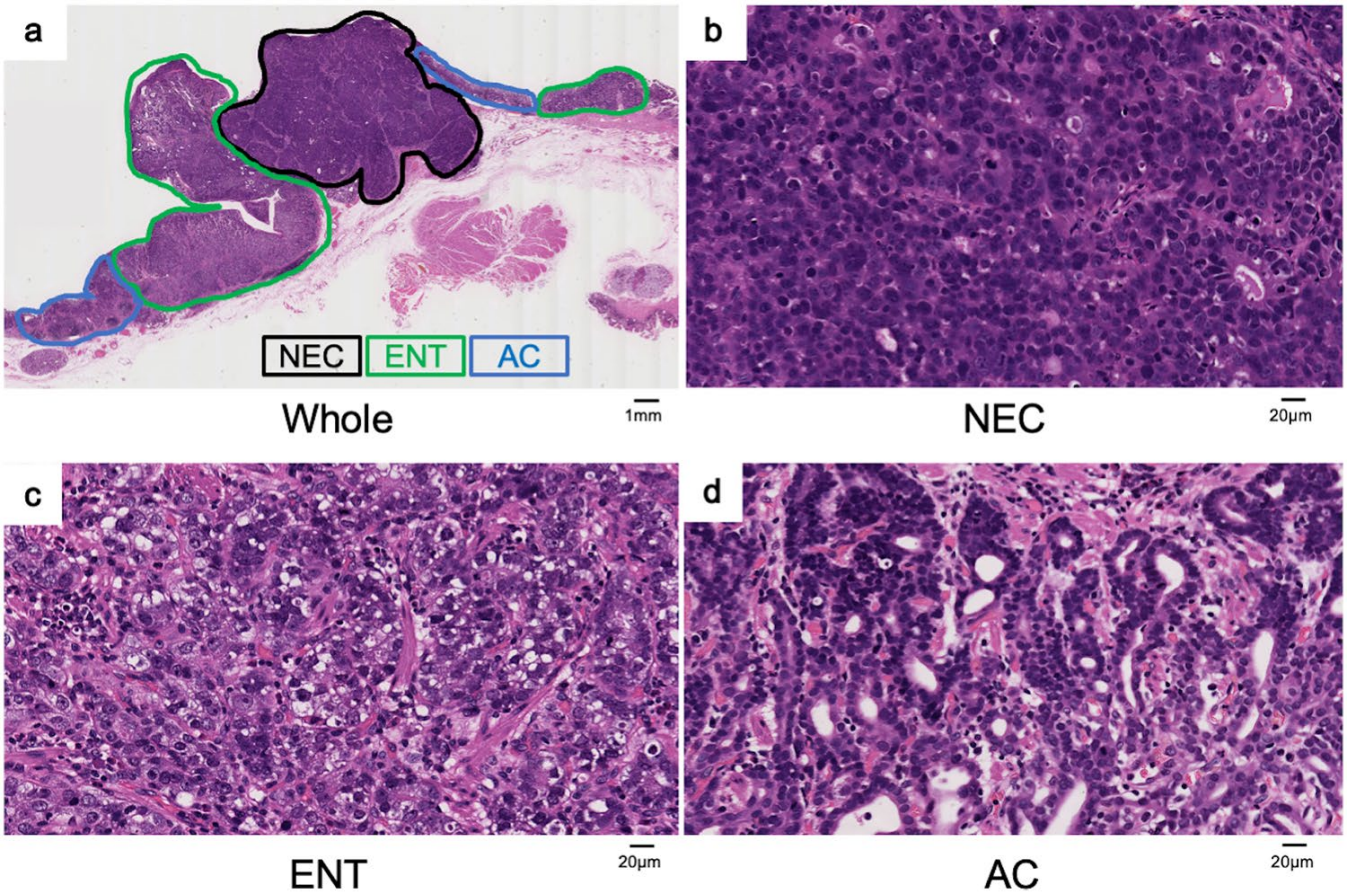
Histological findings of the tumor (Hematoxylin and Eosin staining). **a** The tumor was comprised of three different histological types of carcinoma; **b** neuroendocrine carcinoma (NEC), **c** tumor cells with enteroblastic differentiation (ENT) and **d** moderately differentiated adenocarcinoma (AC)

**Fig. 3:**
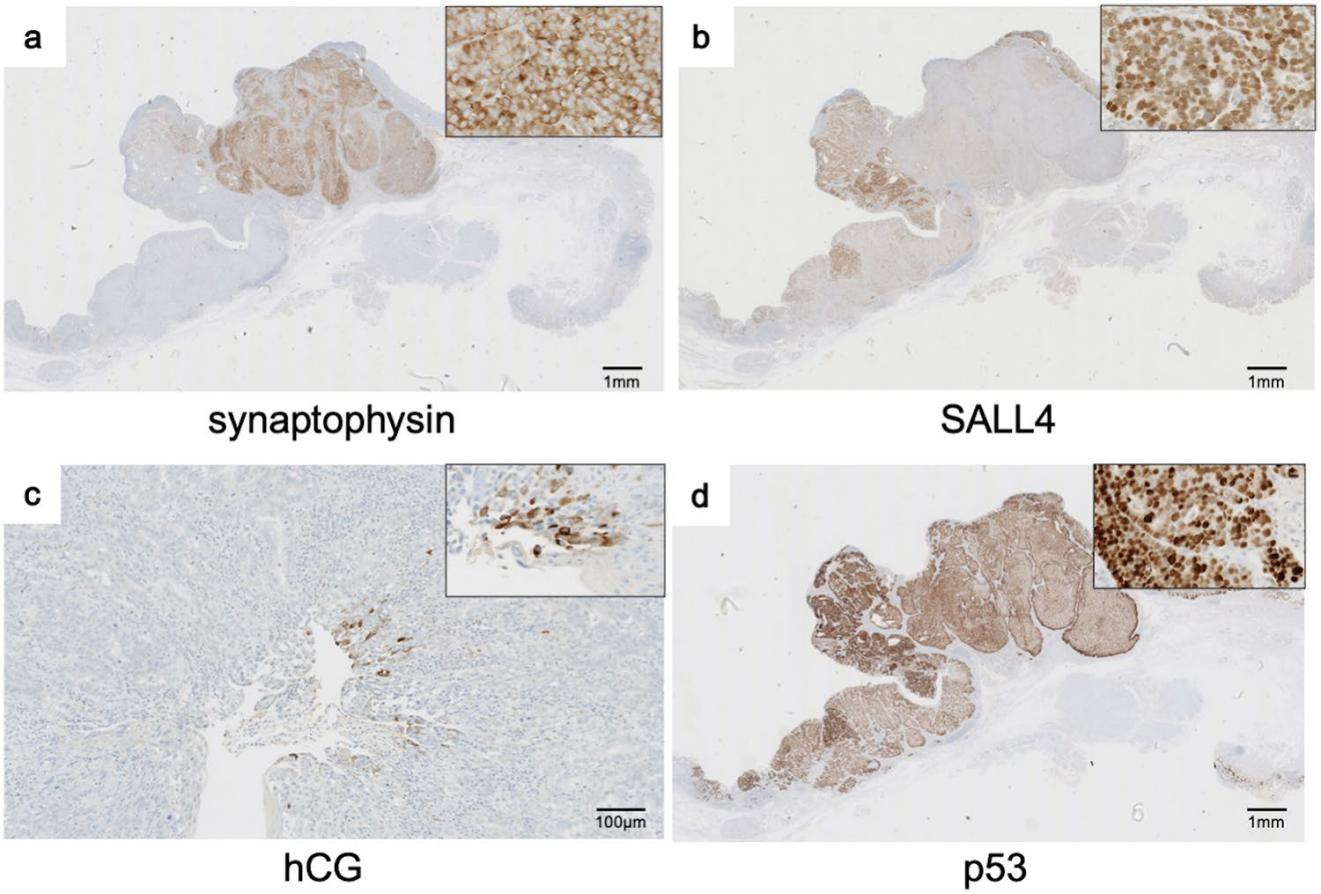
Immunohistochemical findings. **a** NEC was positive for synaptophysin. AC with ENT differentiation was positive for **b** sal-like protein 4 (SALL4) and focally positive for **c** hCG. **d** All parts of the tumor were positive for p53

The tumor was comprised of three different histological types of carcinoma with transitional areas (Fig. 2a), showing characteristic immunohistochemical phenotypes described below. Most of the tumor consisted of the two neoplastic cell types. The first component was round carcinoma cells with round or oval nuclei in a nested or sheeted pattern (Fig. 2b). The tumor cells showed positivity for neuroendocrine markers such as synaptophysin (Fig. 3a), chromogranin A (Supplementary Fig. 1a) and insulinoma-associated protein 1 (INSM1) (Supplementary Fig. 1b) with a Ki-67 index of 60.6%, indicating the cells to be NEC. The second consisted of columnar cells with prominent clear cytoplasm growing in tubular, papillary and solid patterns (Fig. 2c). Immunohistochemical evaluation showed that the cells were immunopositive for sal-like protein 4 (SALL4, a fetal gastrointestinal epithelial marker) (Fig. 3b), focally immunopositive for human chorionic gonadotrophin (hCG) (Fig. 3c), largely immunopositive for AFP (Supplementary Fig. 1c), and partially immunopositive for Glypican3 (Supplementary Fig. 1d), suggesting the cells to exhibit ENT differentiation. The rest consisted of moderately differentiated AC (Fig. 2d). Carcinoma involved the submucosa (sm, 5000 μm from the surface of the lesion).

All parts of the tumor were positive for p53 (Fig. 3d). The AC and the NEC component were both positive for CEA (Supplementary Fig. 2a). Most of the component that was positive for CEA was separate from the CEA-negative ENT component area, though several areas showed positivity for both CEA and AFP (Supplementary Fig. 2b-c).

**Fig. 4:**
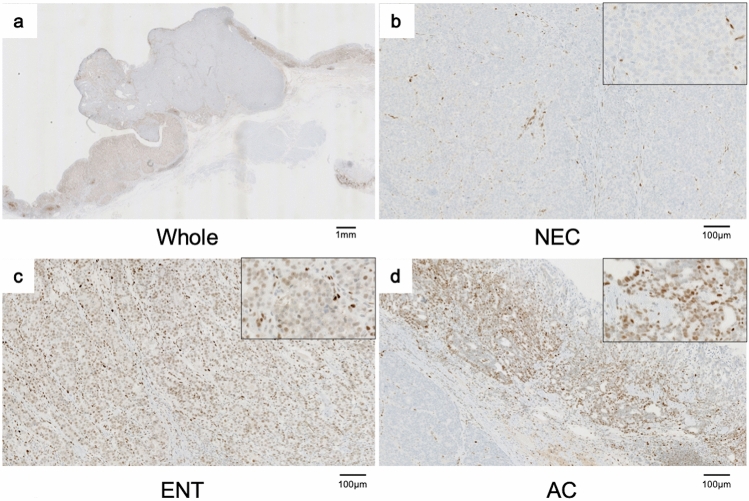
Rb expression. The NEC component was negative, but the AC and ENT components were positive, for retinoblastoma gene protein (Rb)

The amounts of AC, NEC and ENT (including focal hepatoid features and areas of indistinct transition) were approximately 20%, 40% and 40%, respectively. Other immunohistochemical results are summarized in Table 1. Placental alkaline phosphatase, p40 (deltaNp63) and HER2 were negative in all parts of the tumor. Vimentin, a marker for carcinosarcoma, was negative. Ki-67 labeling indexes were 45.8%, 60.6% and 62.4% in the AC, NEC and ENT components, respectively. Mismatch repair proteins were positive in all parts of the tumor. Immunohistochemistry showed that CDX-2 was positive, while MUC-6 and MUC-5AC were negative, suggesting the tumor to be intestinal-type. Retinoblastoma gene protein (Rb) was negative in the NEC, but positive in the AC and ENT (Fig. 4). CD4 and CD8 densities were lower in the NEC segment than in the AC and ENT segments, and programmed cell death ligand-1 (PD-L1) expression was negative in all three components (Supplementary Fig. 3a-d).

**Table 1:** Immunohistochemical evaluation of each histological component

Antibody	Clone	Manufacturer	Results		
			AC	NEC	ENT
Ki-67 index HER2 p53 p40 CDX-2 MUC-6 MUC-5AC Rb HCG PLAP SALL4 AFP Glypican3 Vimentin CEA Synaptophysin INSM1 Chromogranin A PD-L1 (CPS) PD-L1 (TPS) CD4 (/mm^2^) CD8 (/mm^2^) HLA Class1 MLH1 MSH2 MSH6 PMS2	MIB-1 4B5 DO-7 BC28 EPR2764Y CLH5 MRQ-19 EPR17512 Polyclonal 8A9 6E3 polyclonal 1G12 SP20 Polyclonal DAK-SYNAP A8 DAK-A3 22C3 28–8 SP35 C8/144B EMR8-5 EPR3894 3A2B8C EPR3945 A16-4	Dako Roche Dako BioSystems CELLMARQUE Leica Novocastra Abcam Dako Dako Wako DAKO NICHIREI NICHIREI Dako Dako Santa Cruz Dako Dako Dako CELLMARQUE Dako Abcam Abcam Abcam Abcam Roche	45.8 − + − + − − + − − − − − − + − − − 0.36 0.04 3285.8 364.0 + + + + +	60.6 − + − + − − − − − − − − − + + + + 0.79 0.13 1292.2 194.4 + + + + +	62.4 − + − + − − + f+ − + + + − f + − − − 0.76 0.16 3153.3 237.6 + + + + +

*AC* adenocarcinoma, *NEC* neuroendocrine carcinoma, *ENT* adenocarcinoma with enteroblastic differentiation; Rb, retinoblastoma gene protein

+, positive staining, diffusely; f+, positive staining in a small part of the lesion; -, negative staining

There was no evidence of either lymphatic or blood vessel invasion. Nodal metastasis was absent. The tumor was pathologically classified as pT1bN0M0, stage I [9].

## Discussion

Herein, we have demonstrated a very rare case with an early EC arising in BE. The tumor showed a multidirectional differential potential. In our present case, the tumor was comprised of tubular AC, NEC and AC with ENT differentiation. A recent study documented a case of AC with ENT differentiation arising in the ampulla of Vater [10], but only a few reports have described Barrett’s esophageal adenocarcinoma with ENT and NEC components [5]. Notably, most of the previously documented cases had advanced tumors, such that our present case with an early tumor is quite rare in the literature. Furthermore, we conducted detailed analyses on various immunohistochemical markers and tumor immune microenvironments.

Histologically, BE is characterized by proper esophageal glands or ducts beneath the overlying columnar epithelium, squamous epithelial islets in the columnar epithelium, and double-layered lamina muscularis mucosae [11]. In our present case, CDX-2 was strongly positive while MUC-6 and MUC-5AC were negative, a finding in line with previous reports suggesting most Barrett carcinomas to arise from intestinal-type metaplasia [12].

Esophageal NEC is very rare, accounting for approximately 0.2% of all cases of EC [13]. Neuroendocrine neoplasms are diagnosed by immunostaining, such as specific stains for synaptophysin, chromogranin A and INSM1 [14]. Given the high sensitivity of synaptophysin for NEC [15], we identified the NEC as synaptophysin-positive lesions. Recently, INSM1 has emerged as an additional general neuroendocrine marker because it was shown to have better diagnostic ability than synaptophysin and chromogranin A in gastrointestinal tumors [15, 16].

Adenocarcinoma with ENT differentiation arising in BE is also rare. A primitive intestine-like structure, composed of cuboidal or columnar cells with clear cytoplasm, is reportedly a characteristic feature of enteroblastic lesions [6, 17]. Fetal gastrointestinal epithelial markers such as AFP, Glypican 3, and SALL4 have been widely used to immunohistochemically confirm ENT features [6]. In particular, AFP expression is reportedly detectable in nearly half of gastric tumors with ENT differentiation [6]. Motoyama et al. previously reported combined choriocarcinoma, NEC and AC, and detected the expression of hCG in the choriocarcinoma component [5]. In our present case, hCG was focally expressed in the ENT lesion, though the amount was too small for analysis.

We also evaluated the immune microenvironment for each component. Our present case suggested intratumoral CD4 + and CD8 + T cell infiltrations to be modest in NEC as compared to the other components. This observation agrees with a previous study revealing NEC to show low intratumoral CD3 + T cell infiltration [18]. PD-L1 expression was low in all of three histological tumor types observed in our case. A recent study demonstrated PD-L1 expression to be elevated in a high proportion of NEC samples [8], while another study suggested the opposite results [18]. Tumor microenvironment analysis merits further scrutiny in patients with NEC and AC with ENT differentiation.

The carcinogenetic pathway of NEC is not yet fully understood, but two hypotheses have been proposed. First, the originally present malignant exocrine cell develops into a neuroendocrine tumor. Second, monoclonal multi-potent stem cells differentiate into two components. In our present case, both the NEC and AC components were positive for CEA. These observations, taken together with previous findings [19], suggest malignant cells which had originally been directed towards the development of AC to differentiate into NEC. It is noteworthy that Rb expression was absent only in the NEC component in our case. A recent investigation revealed alterations of Rb to be associated with the development of gastrointestinal NEC [20].

Prior studies have revealed AC with ENT differentiation to develop from AC with an intestinal phenotype [17, 21, 22]. In our present case, AFP-positive cells were also observed at the bases of AC tubules, a finding suggesting the origin of ENT to be AC. Furthermore, CEA was focally positive in the ENT lesion. The lesion might have been comprised of tumor cells that partially differentiated into ENT from AC, thereby expressing both CEA and AFP. It is noteworthy that a recent study documented a case which suggested de novo development of gastric AC with ENT differentiation [23]. Further studies are warranted to elucidate the origin, growth, and morphology of this malignancy.

The optimal treatment strategy remains to be determined in esophageal AC, with both NEC and ENT differentiation, due to the rarity of such malignancies. Esophageal NEC, as well as EC with ENT differentiation, is reportedly associated with poor clinical outcomes and high hepatic metastatic potential [7, 24]. Therefore, close follow-up observation is necessary for our present case.

In conclusion, we have described a very rare case with a combination of NEC, AC with ENT differentiation and tubular AC arising in BE. Our present case might contribute to understanding the carcinogenesis, biology and immune microenvironments of such rare malignancies, as well as helping clinicians to manage these complex cases.

## Supplementary Information

Below is the link to the electronic supplementary material.

Supplementary file1 NEC and ENT markers. The NEC component showed positivity for (a) chromogranin A and (b) insulinoma-associated protein 1 (INSM1). The ENT component was (c) largely immunopositive for AFP and (d) partially immunopositive for Glypican3 (TIFF 7298 KB)

Supplementary file2 CEA and AFP expressions. (a) Both the adenocarcinoma and the neuroendocrine component stained positive for CEA. (b-c) Several areas were positive for both CEA and AFP (TIFF 7473 KB)

Supplementary file3 CD4, CD8 and PD-L1 status according to the histological type. (a-c) CD4 and (d-f) CD8 densities were lower in the NEC segment than in the AC and ENT segments, and (g-i) all three components were negative for PD-L1 expression (TIFF 6671 KB)

## Abbreviations

BE: Barrett’s esophagus
EC: Esophageal carcinoma
NEC: Neuroendocrine carcinoma
ENT: Enteroblastic
AFP: Alpha-fetoprotein
CEA: Carcinoembryonic antigen
AC: Adenocarcinoma
INSM1: Insulinoma-associated protein 1
hCG: Human chorionic gonadotrophin
SALL4: Sal-like protein 4
Rb: Retinoblastoma gene protein
PD-L1: Programmed cell death ligand-1
