# Supplementary figures and images for: Combined tubular adenocarcinoma, neuroendocrine carcinoma and adenocarcinoma with enteroblastic differentiation arising in Barrett esophagus
Source: Clin J Gastroenterol. 2023 Apr 7;16(4):501–7. doi: 10.1007/s12328-023-01791-0 (PMC10356886; doi:10.1007/s12328-023-01791-0)

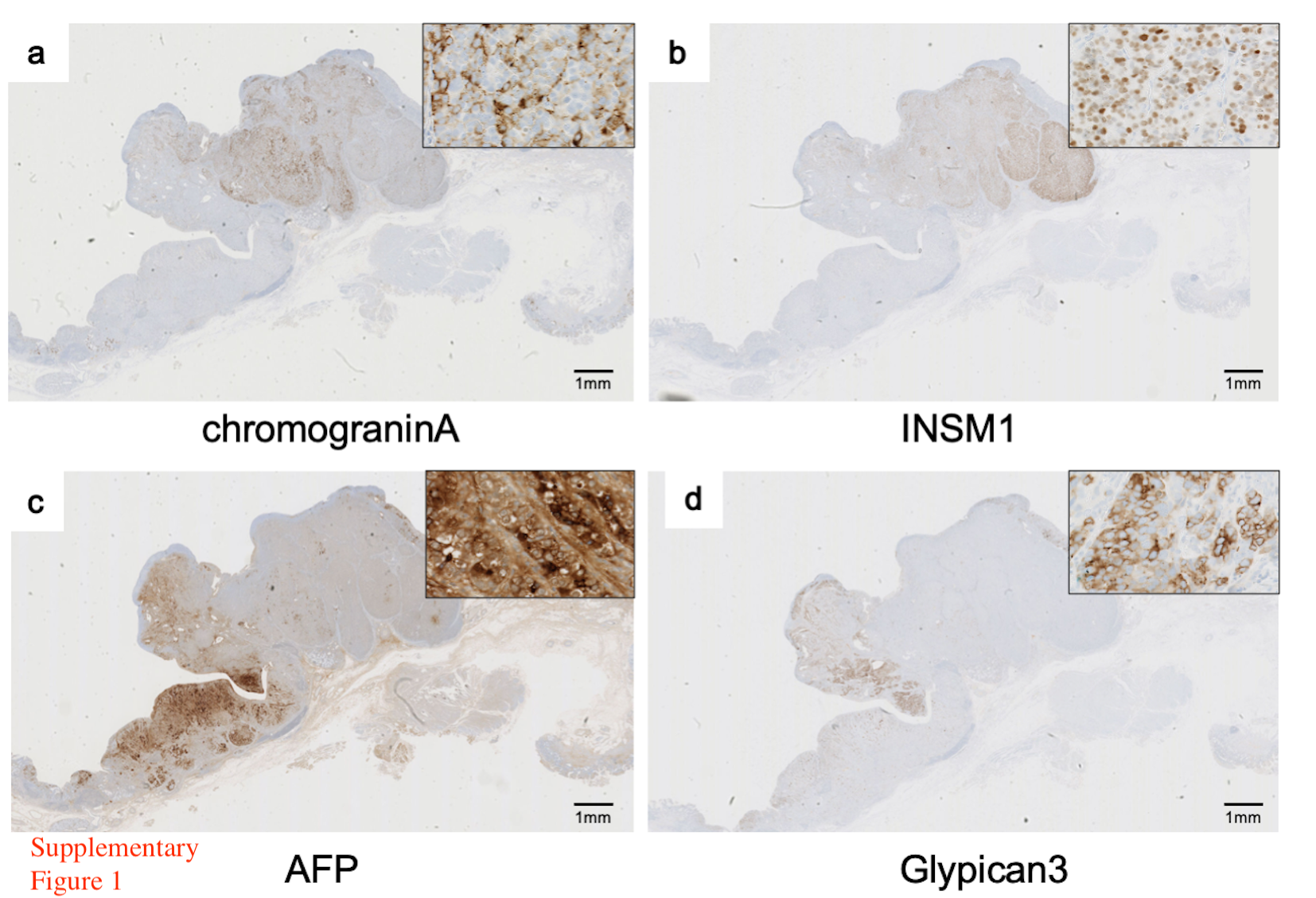

Supplement: Supplementary file 1 — Supplementary file1 NEC and ENT markers. The NEC component showed positivity for (a) chromogranin A and (b) insulinoma-associated protein 1 (INSM1). The ENT component was (c) largely immunopositive for AFP and (d) partially immunopositive for Glypican3 (TIFF 7298 KB) [file 12328_2023_1791_MOESM1_ESM.tiff]

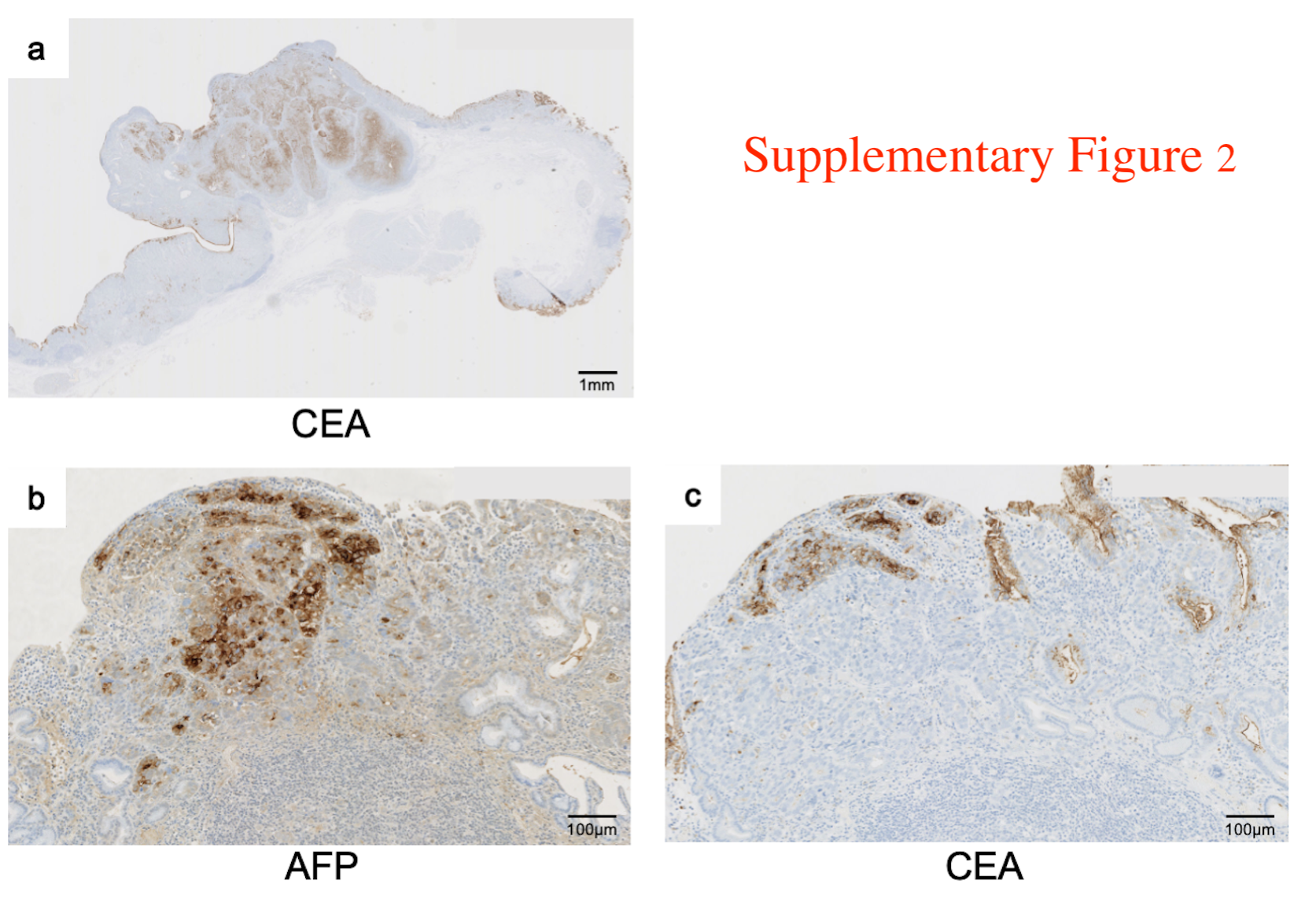

Supplement: Supplementary file 2 — Supplementary file2 CEA and AFP expressions. (a) Both the adenocarcinoma and the neuroendocrine component stained positive for CEA. (b-c) Several areas were positive for both CEA and AFP (TIFF 7473 KB) [file 12328_2023_1791_MOESM2_ESM.tiff]

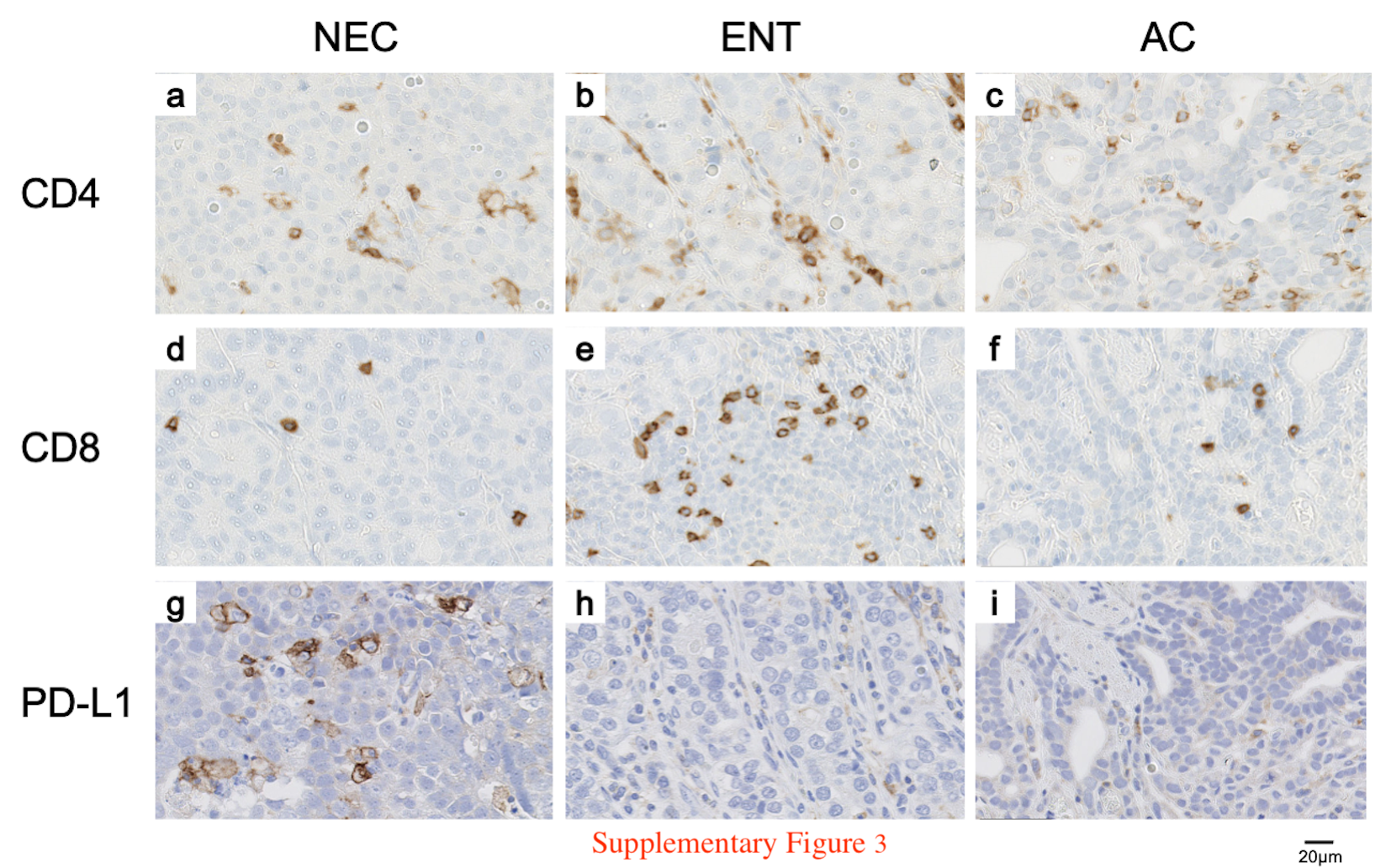

Supplement: Supplementary file 3 — Supplementary file3 CD4, CD8 and PD-L1 status according to the histological type. (a-c) CD4 and (d-f) CD8 densities were lower in the NEC segment than in the AC and ENT segments, and (g-i) all three components were negative for PD-L1 expression (TIFF 6671 KB) [file 12328_2023_1791_MOESM3_ESM.tiff]
